# Design and structural characterisation of olfactomedin-1 variants as tools for functional studies

**DOI:** 10.1186/s12860-019-0232-1

**Published:** 2019-11-14

**Authors:** Matti F. Pronker, Hugo van den Hoek, Bert J. C. Janssen

**Affiliations:** 1 MRC Laboratory of Molecular Biology, Division of Neurobiology, Francis Crick Avenue, Cambridge, CB2 0QH UK; 2 Bijvoet Center for Biomolecular Research, Utrecht University, Crystal and Structural Chemistry, Kruytgebouw, Padualaan 8, 3584 CH Utrecht, The Netherlands; 3 Department of Molecular Structural Biology, Max Planck institute for Biochemistry, Am Klopferspitz 18, 82152 Martinsried, Germany

**Keywords:** Nervous system, Signalling, Synapse, Protein purification, X-ray crystallography, Small-angle X-ray scattering

## Abstract

**Background:**

Olfactomedin-1 (Olfm1; also known as Noelin or Pancortin) is a highly-expressed secreted brain and retina protein and its four isoforms have different roles in nervous system development and function. Structural studies showed that the long Olfm1 isoform BMZ forms a disulfide-linked tetramer with a V-shaped architecture. The tips of the Olfm1 “V” each consist of two C-terminal β-propeller domains that enclose a calcium binding site. Functional characterisation of Olfm1 may be aided by new biochemical tools derived from these core structural elements.

**Results:**

Here we present the production, purification and structural analysis of three novel monomeric, dimeric and tetrameric forms of mammalian Olfm1 for functional studies. We characterise these constructs structurally by high-resolution X-ray crystallography and small-angle X-ray scattering. The crystal structure of the Olfm1 β-propeller domain (to 1.25 Å) represents the highest-resolution structure of an olfactomedin family member to date, revealing features such as a hydrophilic tunnel containing water molecules running into the core of the domain where the calcium binding site resides. The shorter Olfactomedin-1 isoform BMY is a disulfide-linked tetramer with a shape similar to the corresponding region in the longer BMZ isoform.

**Conclusions:**

These recombinantly-expressed protein tools should assist future studies, for example of biophysical, electrophysiological or morphological nature, to help elucidate the functions of Olfm1 in the mature mammalian brain. The control over the oligomeric state of Olfm1 provides a firm basis to better understand the role of Olfm1 in the (trans-synaptic) tethering or avidity-mediated clustering of synaptic receptors such as post-synaptic AMPA receptors and pre-synaptic amyloid precursor protein. In addition, the variation in domain composition of these protein tools provides a means to dissect the Olfm1 regions important for receptor binding.

## Background

**Fig. 1:**
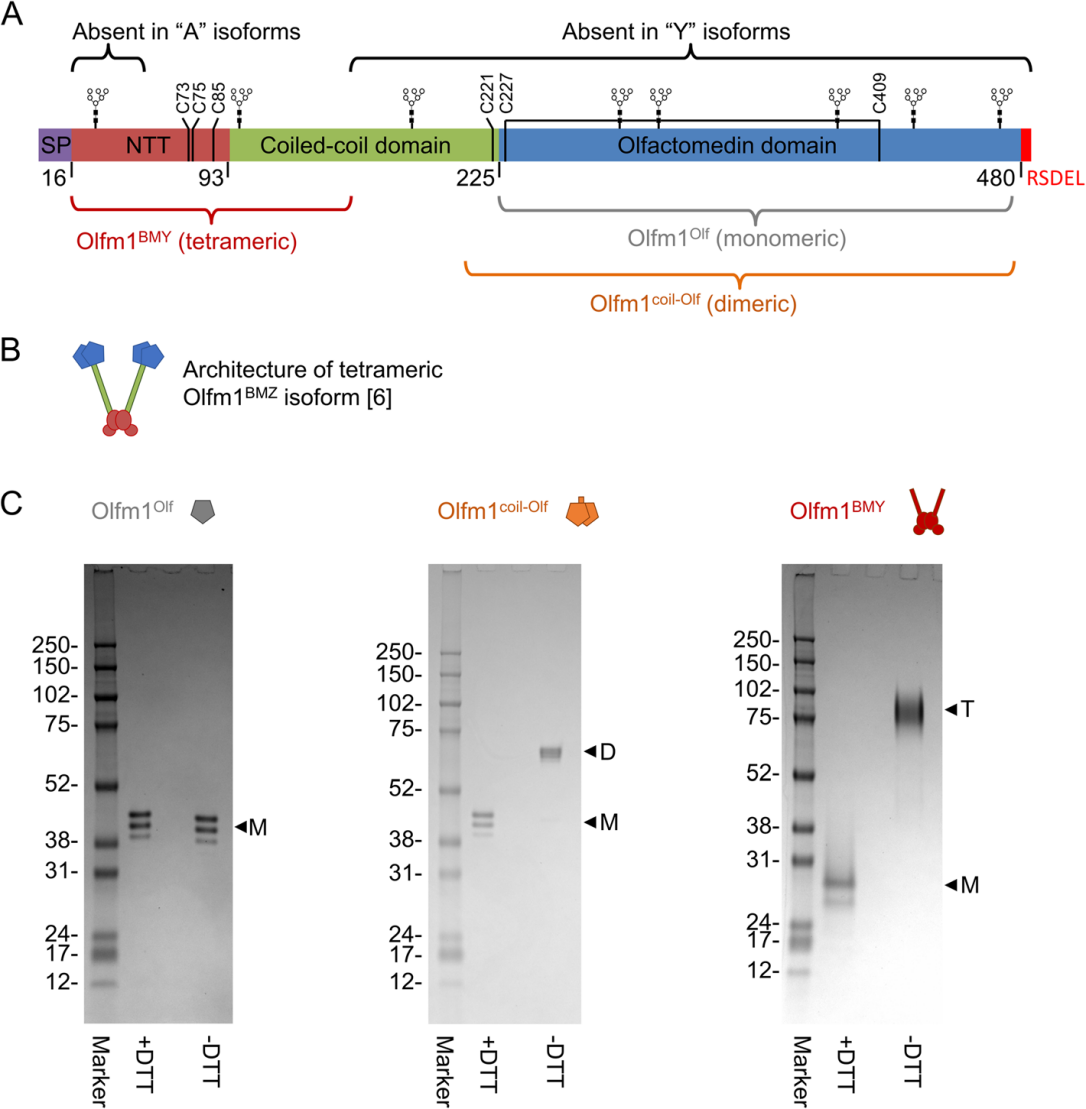
Purification of three novel Olfm1 constructs; Olfm1^Olf^, Olfm1^coil-Olf^ and Olfm1^BMY^. **a** Overview of Olfm1 sequence and isoform differences. Indicated domain widths scale with amino acid sequence length. N-linked glycosylation sites and cysteines are indicated. **b** Architecture of tetrameric Olfm1^BMZ^ (full-length) isoform as previously determined [6]. Colours correspond to domains as indicated in panel a. **c** Coomassie-stained SDS-PAGE analysis of purified Olfm1^Olf^, Olfm1^coil-Olf^ and Olfm1^BMY^ shows a high degree of purity and confirms correct formation of disulfides in Olfm1^coil-Olf^ and Olfm1^BMY^ as evidenced by the shift under non-reducing (−DTT) conditions. The multiple bands predominantly visible in the reduced samples, with apparent weight differences of a few kDa, are the result of heterogeneous N-linked glycosylation (Fig. 2)

Olfactomedin family proteins play important roles in nervous system function and development throughout the animal kingdom [1, 2]. The prototypical member Olfm1 is a secreted glycoprotein expressed at high levels in the brain [3, 4]. As a result of alternative promotor usage and splicing, Olfm1 exists in four isoforms [5], referred to as AMY, BMY, AMZ and BMZ. The letters A, B, M, Y and Z refer to the different exons in each isoform in corresponding sequential order from N- to C-terminus (Fig. 1). These isoforms are differentially expressed in discrete brain regions and over the course of development [5].

The four isoforms share the M exon, which contains a central tetramerisation domain (denoted N-terminal tetramerisation, NTT) [6] that has not been structurally characterised. Otherwise the isoforms differ at the N- and C-termini. The A isoforms have an alternative signal peptide but no unique residues in the mature protein and represent a truncation at the N-terminus of a contiguous stretch of 34 residues compared to the B isoforms. C-terminal of the NTT is a parallel dimeric coiled-coil segment. Y isoforms end with a single glycine residue (Gly153) at the C-terminus of this coiled coil segment (Fig. 1). The longer Z isoforms on the other hand have a more extended coiled coil (69 residues longer), followed by a highly conserved β-propeller domain (residues 226–478) at the C-terminus that represents more than half the protein mass (Fig. 1). These Z isoforms are substantially more abundant in the brain than the shorter Y isoforms [7, 8]. The β-propellers in the Z isoforms are covalently dimerised by an inter-chain disulfide bond (formed by Cys221-Cys221) at the C-terminus of the preceding coiled-coil domain [6]. It is likely that all four isoforms of Olfm1 form disulfide-linked tetramers by disulfide bonds in the NTT domain that they have in common and that this tetrameric nature is important for the function of Olfm1, for example by allowing Olfm1 to cluster multiple cell-surface receptors.

Although the exact functions of Olfm1 in the brain are not understood at the mechanistic level, several studies have found roles for Olfm1 in nervous system development of different vertebrates. Olfm1 was found to stimulate neurogenesis [9], play a role in neural crest generation [10] and stimulate axonal elongation [11]. However, expression of Olfm1 still increases strongly from embryonic and juvenile stages into adulthood in mice [7, 12], suggesting Olfm1 additionally functions in the adult brain beyond its developmental roles.

Olfm1 has been reported to interact with various nervous system cell surface receptors such as Amyloid Precursor Protein (APP) [13], the Nogo Receptor [14], and glutamate-gated ion channels of the α-amino-3-hydroxy-5-methyl-4-isoxazolepropionic acid (AMPA) receptor family GluA1–4 [8, 15–24]. In recent years it was found that Olfm1 is enriched in synapses [8, 22] and proximity labelling identified its presence in the synaptic cleft [25, 26]. Taken together the findings that Olfm1 is secreted [4, 9, 10], interacts with synaptic cell surface receptors, is enriched in the synapse and is present in the synaptic cleft suggest that Olfm1 plays a role in this intercellular substructure.

In line with this hypothesis, a mutation that results in deletion of 52 residues in the coiled coil region of Olfm1 leads to brain dystrophy, altered interaction with synaptic components and aberrant calcium signalling and behaviour in mice [18], as well as functional deficits of the eye [27]. A full knockout of Olfm1 in zebrafish showed impaired AMPA receptor trafficking and reduced levels of pre- and post-synaptic proteins such as VAMP-2 and GluA2 [22]. Finally, a recent study showed that Olfm1 decreases surface mobility of synaptic AMPA receptors [8], directly linking Olfm1 to controlling synaptic plasticity [28]. However, the exact role and the mechanisms by which Olfm1 exerts those functions are yet to be determined.

In recent years, structures of the Olf domain of a number of olfactomedin family members have been determined, such as those of gliomedin, myocilin, latrophilin-3 and Olfm1 itself [6, 29–36]. Although progress has been made towards elucidating the functions of Olfm1 in the (mature) brain, studies are hampered by a lack of molecular tools to dissect Olfm1 interactions at the mechanistic level. Here we describe new recombinant Olfm1 constructs and purification strategies to obtain pure monomeric, dimeric and tetrameric variants of mammalian Olfm1 that can be used for functional studies. We characterise the structures of these constructs by high-resolution X-ray crystallography and small-angle X-ray scattering (SAXS). The structural data indicate the samples are of high quality and suitable as molecular probes to study Olfm1 function. Moreover, our structures reveal new insights such as a hydrophilic tunnel containing water molecules running into the hydrophobic core of the C-terminal β-propeller domain, that connects to the sodium and calcium binding sites. We also confirm that, similar to other Olf family members [29–35], the bound Na^+^ and Ca^2+^ ions stabilise a surface loop at the top face of the β-propeller. Finally, we show that the shorter BMY isoform forms disulfide-linked tetramers, consistent with the architecture of the longer BMZ isoform [6].

## Materials and methods

### Constructs

All constructs were obtained via polymerase chain reaction using *Mus musculus* (mouse) Olfm1 BMZ isoform (NCBI Reference Sequence NP_062371) cDNA IRAVp968C0174D (Source Bioscience) as a template. They were subsequently subcloned using BamHI and NotI restriction sites into the pUPE107.03 (U-Protein Express) mammalian expression vector containing an Epstein-Barr virus origin of replication, a C-terminal His_6_-tag and a cystatin signal peptide for secretion. Residues (UNIPROT numbering) 226–478 (Olfm1^Olf^), 212–478 (Olfm1^coil-Olf^) or 17–153 (Olfm1^BMY^; residue 153 being a glycine as in native BMY), are flanked by an N-terminal GS- and a C-terminal -AAAHHHHHH sequence in the mature protein as a result of the restriction sites and affinity tag. The C-terminal -VIRSDEL residues have not been included in the Olfm1^Olf^ and Olfm1^coil-Olf^ constructs as removal enhanced expression and secretion levels.

### Protein expression and purification

Constructs were transiently transfected with polyethylenimine in suspension culture growing *N*-acetylglucosaminyltransferase I-deficient (GntI−/−) Epstein-Barr virus nuclear antigen I-expressing HEK293 cells (U-Protein Express) in Freestyle™ medium, following established protocols [37]. After 6 days, cell supernatant was harvested by centrifugation at 1000×g for 15 min and filtered through a 0.22 μm filter. The filtered supernatant was concentrated fivefold and buffer exchanged to 500 mM NaCl, 5 mM CaCl_2_, 25 mM HEPES pH 7.8, (IMAC A buffer) using a 10 kDa molecular weight cut-off (MWCO) membrane. Protein was purified by nickel-nitrilotriacetic acid affinity chromatography, using a pre-packed Histrap column (GE Healthcare). For Olfm1^Olf^, the column was washed with 20 column volumes of IMAC A supplemented with 40 mM imidazole and eluted with IMAC A supplemented with 200 mM imidazole (pH adjusted to pH 7.8 after adding imidazole). Due to their predicted oligomeric nature, washing and elution were performed with higher concentrations of imidazole for Olfm1^coil-Olf^ and Olfm1^BMY^; washing with IMAC A supplemented with 50 mM imidazole (for 20 column volumes) and eluting with 500 mM imidazole in IMAC A.

**Fig. 2:**
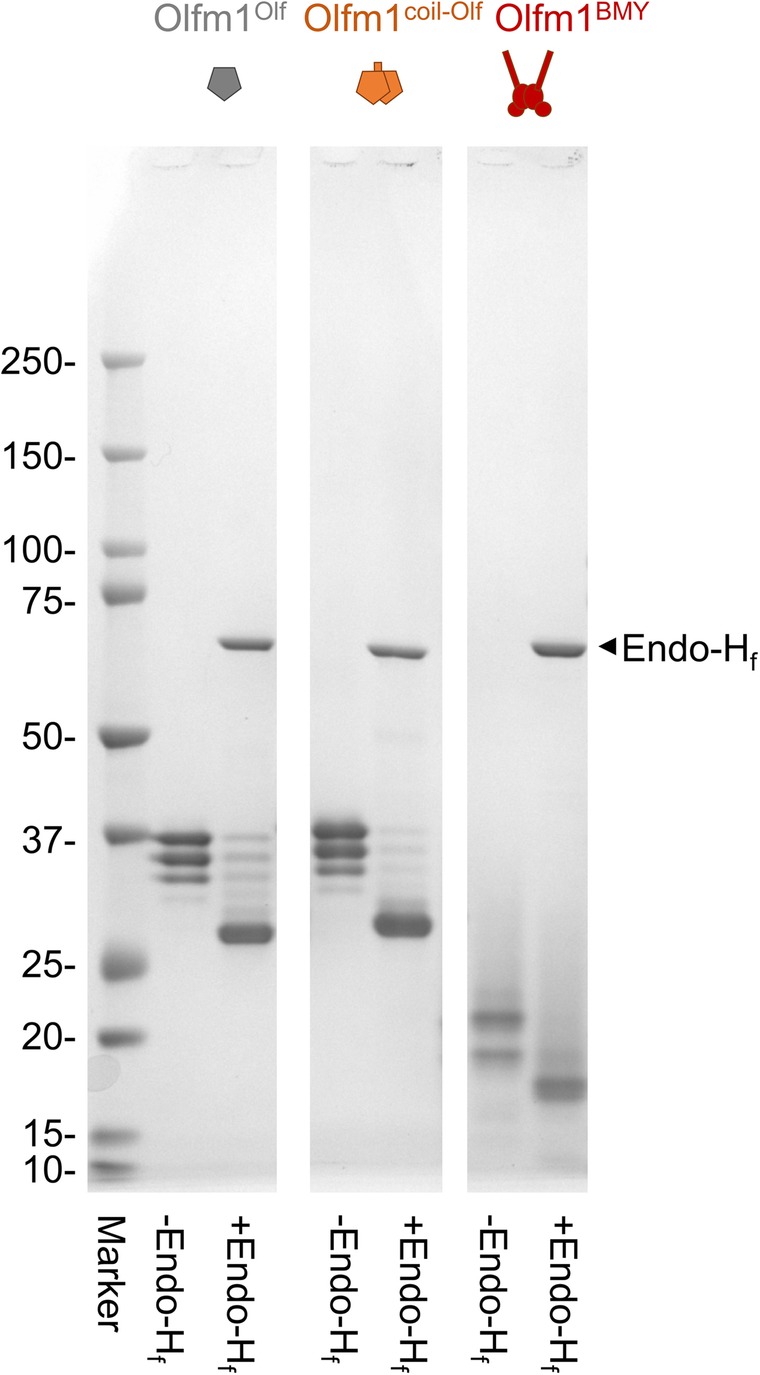
Glycosylation of Olfm1^Olf^, Olfm1^coil-Olf^ and Olfm1^BMY^ is heterogeneous. Band multiplets visual in reduced SDS-PAGE are the result of heterogeneous glycosylation. Deglycosylation of Olfm1^Olf^, Olfm1^coil-Olf^ and Olfm1^BMY^ by Endo-H_f_ under denaturing condition reduces the heterogeneity; the multiplets, visible in the untreated samples, disappear and a prominent single band of lower molecular mass remains

The eluate was concentrated using 10 kDa MWCO centrifugal filter units (Amicon®). Subsequent purification was performed by size exclusion chromatography (SEC) on a Superdex75 Hiload 16/60 column (for Olfm1^Olf^) or a Superdex200 Hiload 16/60 column (for Olfm1^coil-Olf^ and Olfm1^BMY^) (GE Healthcare), equilibrated in SEC buffer (150 mM NaCl, 2 mM CaCl_2_, 20 mM HEPES, pH 7.5). Protein purity, covalent oligomeric state and glycosylation state were assessed by SDS-PAGE (Figs. 1 and 2).

**Fig. 3:**
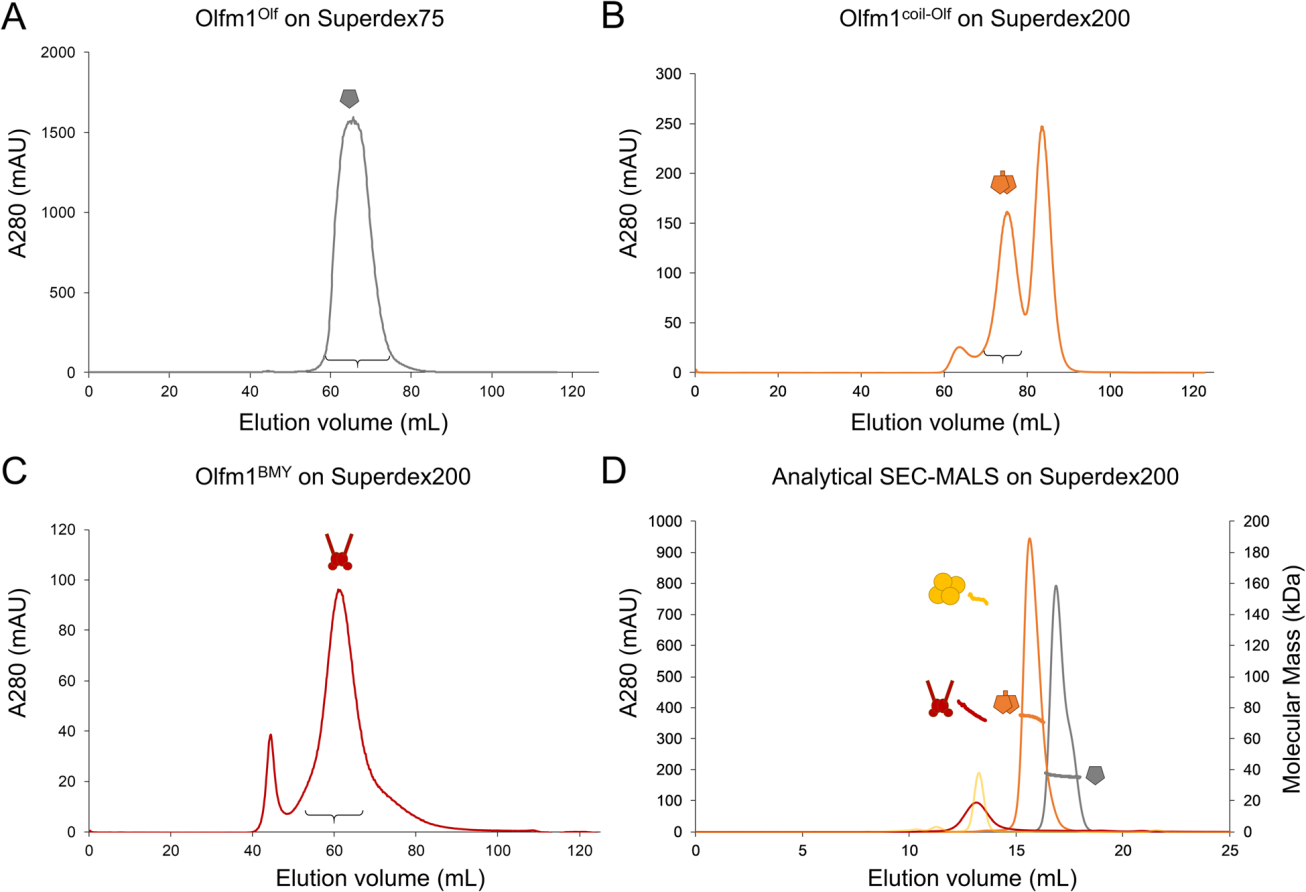
Preparative and analytical SEC profiles of the three Olfm1 constructs confirm their predicted oligomeric state **a** SEC chromatogram of Olfm1^Olf^ on a HiLoad 16/60 Superdex75 column. **b** SEC chromatogram of Olfm1^coil-Olf^ on a HiLoad 16/60 Superdex200 column, corresponding to the second injection (see Materials and methods section for details). **c** SEC chromatogram of Olfm1^BMY^ on a HiLoad 16/60 Superdex200 column. An accolade indicates the pooled fractions for all three preparative chromatograms. **d** Analytical SEC-MALS analysis on a Superdex200 10/300 increase column of Olfm1^Olf^, Olfm1^coil-Olf^ and Olfm1^BMY^ confirms their predicted respective monomeric, dimer and tetrameric states (colours corresponding to panels a, b and c, respectively). The standard, aldolase (158 kDa), is shown in yellow and was used to calibrate the MALS signal

For Olfm1^coil-Olf^, peak fractions containing only disulfide-linked dimer according to non-reducing SDS-PAGE were pooled. Fractions containing disulfide-linked dimer contaminated with monomer were subjected to a second step of SEC on the same column, allowing more correctly folded disulfide-linked dimer to be recovered (Figs. 1c and 3b). Protein was concentrated to 14.3 mg/mL (Olfm1^Olf^) or 6 mg/mL (Olfm1^coil-Olf^ and Olfm1^BMY^) using 10 kDa MWCO centrifugal filter units (Amicon®) before aliquoting and plunge-freezing in liquid nitrogen.

### Deglycosylation

To test whether the observed heterogeneity (multiplets) on reducing SDS-PAGE (Fig. 1c) was caused by heterogeneous glycosylation, we performed deglycosylation with Endo-H_f_ (New England Biolabs) under denaturing conditions. Samples were heated to 368 K for 10 min in denaturing buffer (provided with the enzyme) before letting them cool down to 298 K and adding Endo-H_f_ at a ratio of 1:10 (v/v). The reaction was incubated overnight at 298 K, before performing SDS-PAGE under reducing conditions together with untreated sample at the same concentration (Fig. 2).

### SEC-MALS

Size-exclusion chromatography with multi-angle light scattering (SEC-MALS) was performed at room temperature using an analytical Superdex200 Increase 10/300 column (GE Healthcare) equilibrated with SEC buffer. SEC was performed with online static light scattering (miniDAWN TREOS, Wyatt Technology) and differential refractive index (dRI, Shimadzu RID-10A) on a Shimadzu HPLC system equipped with a temperature-controlled autosampler (SIL-20 AC, at 277 K) and column oven (CTO-20 AC; at 293 K), at a flowrate of 0.5 mL/min. Data were analysed using the ASTRA software suite (Wyatt Technology). The dRI signal was combined with the light scattering to determine the molecular mass using standard protocols. A dn/dc of 0.178 mL/g was used for Olfm1^Olf^, and of 0.180 mL/g for Olfm1^coil-Olf^ and Olfm1^BMY^, based on the number of N-linked glycans. Rabbit Aldolase was injected at 1 mg/mL as a control and calibration standard (for Aldolase a dn/dc of 0.186 mL/g was used).

### Crystallisation and structure determination

Prior to crystallization, samples of Olfm1^Olf^ and Olfm1^coil-Olf^ were deglycosylated by treatment with Endoglycosidase-H (Endo-H), added at a 1:100 (v/v) ratio and incubating at 37 °C overnight. Crystallization was performed using the sitting drop vapour diffusion method, mixing 150 nL protein sample with 150 nL reservoir solution, at 293 K for Olfm1^Olf^ and at 277 K for Olfm1^coil-Olf^.

Crystals grew in a condition containing 8% (w/v) PEG 8000 and 0.1 M Tris-HCl pH 8.5 for Olfm1^Olf^ and 0.08 M Magnesium Acetate, 30% (w/v) PEG 4000 and 0.05 M Sodium Cacodylate pH 6.5 for Olfm1^coil-Olf^. Crystals were cryo-protected with reservoir solution supplemented with 25% (v/v) glycerol before plunge-cooling in liquid nitrogen. Datasets were collected at 100 K at European Synchrotron Radiation Facility (ESRF) beamline ID30A-3 (Massif-3) for Olfm1^Olf^ or Diamond Light Source (DLS) beamline I03 for Olfm1^coil-Olf^. Grid scanning was used for the Olfm1^Olf^ crystals to find the best-diffracting sub-volume of each crystal.

Data were integrated and scaled by XDS [38] and merged and truncated by the Aimless pipeline [39], respectively. Structures were solved by molecular replacement with PDB 5AMO [6] as a search model using Phaser [40]. Iterative cycles of manual model building in Coot [41] and reciprocal space refinement with Phenix [42] were performed for final refinement. C_α_ RMSDs were calculated by secondary structure matching using the program superpose [43].

### Small-angle X-ray scattering

SAXS was performed at the ESRF BM29 BioSAXS beamline equipped with a 2D Pilatus 1 M detector (DECTRIS, Switzerland) operated at an energy of 12.5 keV. Olfm1^BMY^ was diluted with and dialysed against SEC buffer using a 10 kDa MWCO membrane. The concentration of Olfm1^BMY^ was determined by UV spectrophotometry at 280 nm wavelength on a nanodrop ND-1000 spectrophotometer to be 0.615 mg/mL. SAXS data were collected at 277 K. Ten successive 1.0 s frames were collected. The data were radially averaged and normalised to the intensity of the transmitted beam, exposure time, and sample concentration, and the scattering of the solvent blank (SEC buffer) was subtracted. The curve was scaled to absolute values using a water reference so that the *I*_0_ represents the Olfm1 molecular weight. Radiation damage was monitored by comparing curves collected on the same sample; no evidence for radiation damage was observed. Data were analysed by the Atsas suite [44] programs Primus [45] for the Guinier analysis, Gnom [46] for the pair-distance distribution function and Dammif [47] for the ab-initio modelling.

## Results

We produced monomeric, dimeric and tetrameric variants of *Mus musculus* (mouse) Olfm1 in mammalian cells. The monomeric construct comprises the C-terminal Olfactomedin domain (henceforth referred to as Olfm1^Olf^) (Fig. 1a). The dimeric construct is composed of the Olfactomedin domain and additionally includes part of the coiled coil and the inter-chain disulfide formed by Cys221 at the N-terminus of the Olf domain, similar to our previously crystallised limited proteolysis fragment (Olfm1^coil-Olf^) [6] (Fig. 1a). The third construct corresponds to the natural isoform BMY (Olfm1^BMY^) and therefore lacks the C-terminal half of the coiled coil and the Olfactomedin domains (Fig. 1a), but does include the NTT domain and is thus expected to be tetrameric like the full-length BMZ isoform [6].

All constructs were purified from the supernatant of over-expressing HEK293 GntI−/− cells by a combination of Ni^2+^-affinity chromatography and SEC (Figs. 1c and 3). Care was taken to always include calcium chloride in the purification buffers as we previously observed this profoundly stabilises full-length Olfm1^BMZ^ [6]. Higher concentrations of imidazole were used for washing and eluting the dimeric Olfm1^coil-Olf^ and tetrameric Olfm1^BMY^ than for the monomeric Olfm1^Olf^ (see Materials and methods section for details) as they are expected to have two and four tags per molecule, respectively. We would like to remark that the inclusion of calcium chloride in buffers and the washing and elution with higher concentrations of imidazole (50 instead of 40 mM for washing and 500 instead of 200 mM for elution) also improve the yield and purity of tetrameric Olfm1^BMZ^ (data not shown) compared to our previously-published tetrameric Olfm1^BMZ^ purification strategy [6]. Predicted oligomeric state of the three constructs was confirmed by analytical SEC-MALS (Fig. 3d), revealing molecular masses of 36 ± 1 kDa for Olfm1^Olf^ (35 kDa is predicted for a monomer including 4 N-linked glycans), 73 ± 1 kDa for Olfm1^coil-Olf^ (73 kDa is predicted for a dimer including 8 N-linked glycans) and 77 ± 4 kDa for Olfm1^BMY^ (77 kDa is predicted for a tetramer including 8 N-linked glycans).

### Monomeric Olfm1^Olf^

Based on our previous structure [6], we designed truncations that are expected to result in a monomeric domain by truncating the dimerizing coiled coil and excluding Cys221 from the construct (Fig. 1, the Olfm1^Olf^ construct includes UNIPROT residues 226–478). This construct expressed with high yields in HEK293 cells (about 30 mg from a litre of HEK293 suspension cell culture) and could be purified using standard protocols (see Materials and methods section for details).

**Fig. 4:**
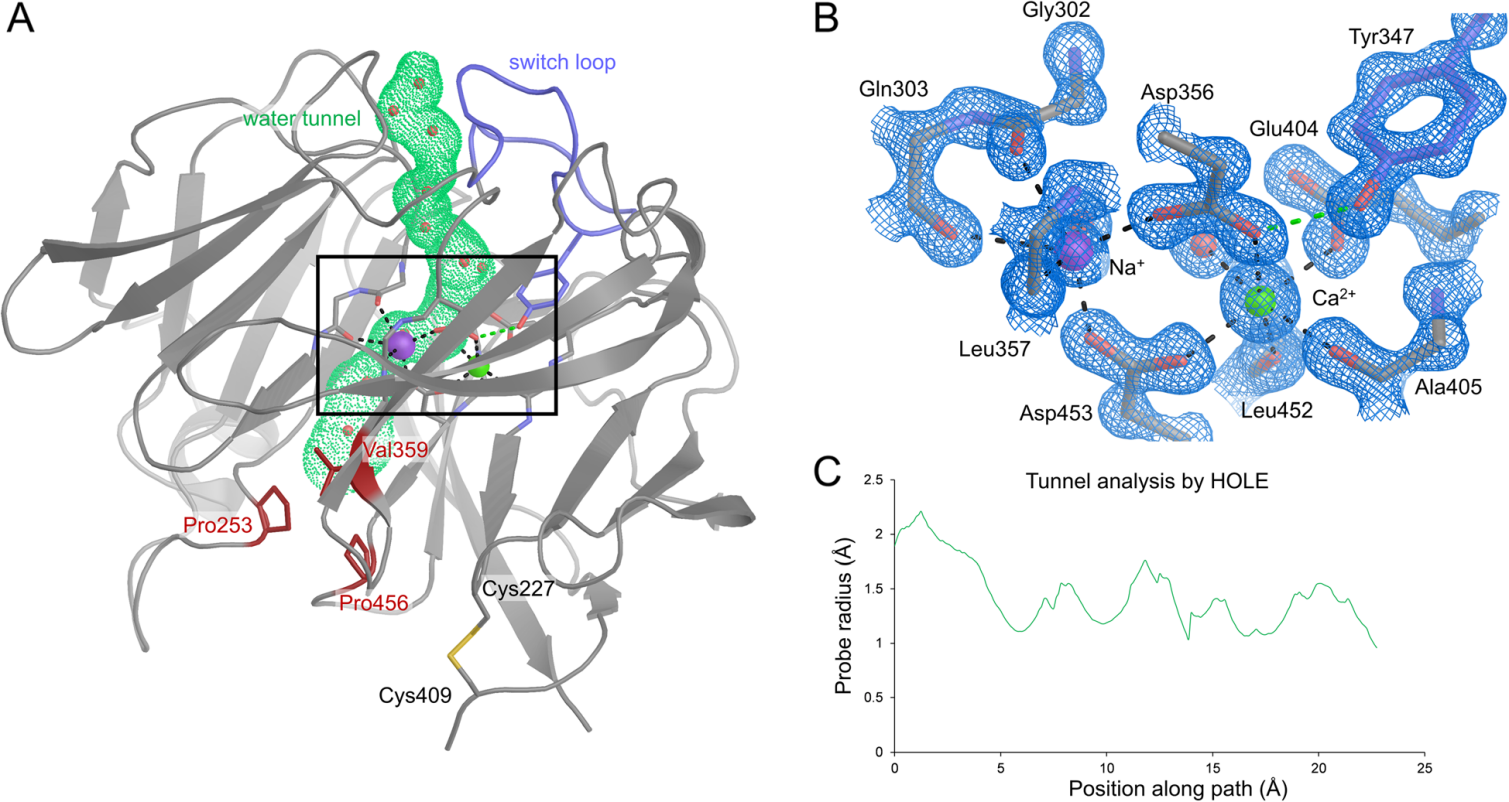
High-resolution crystal structure of Olfm1^Olf^ to 1.25 Å with bound Na^+^ and Ca^2+^ ions reveals a structured switch loop and a water tunnel running to the metal ion binding sites. **a** Overview of the Olfm1^Olf^ β-propeller domain with the bound Na^+^ (purple) and Ca^2+^ (green) ions. The switch loop is indicated in violet, the water tunnel in green, the hydrophobic plug residues closing the tunnel in dark red and individual water molecules in the tunnel are represented as red spheres. The intra-chain disulfide between Cys227 and Cys409 is shown in sticks representation. **b** Close-up of the metal ion binding site with 2F_o_-F_c_ electron density contoured at 2.5 σ. Metal ion-coordinating interactions are shown as black dashes and the hydrogen bond of the coordinating carboxylic acid group of Asp356 with the hydroxyl group of Tyr347 in the switch loop is indicated in green. **c** Analysis of the tunnel radius by HOLE [48]

**Fig. 5:**
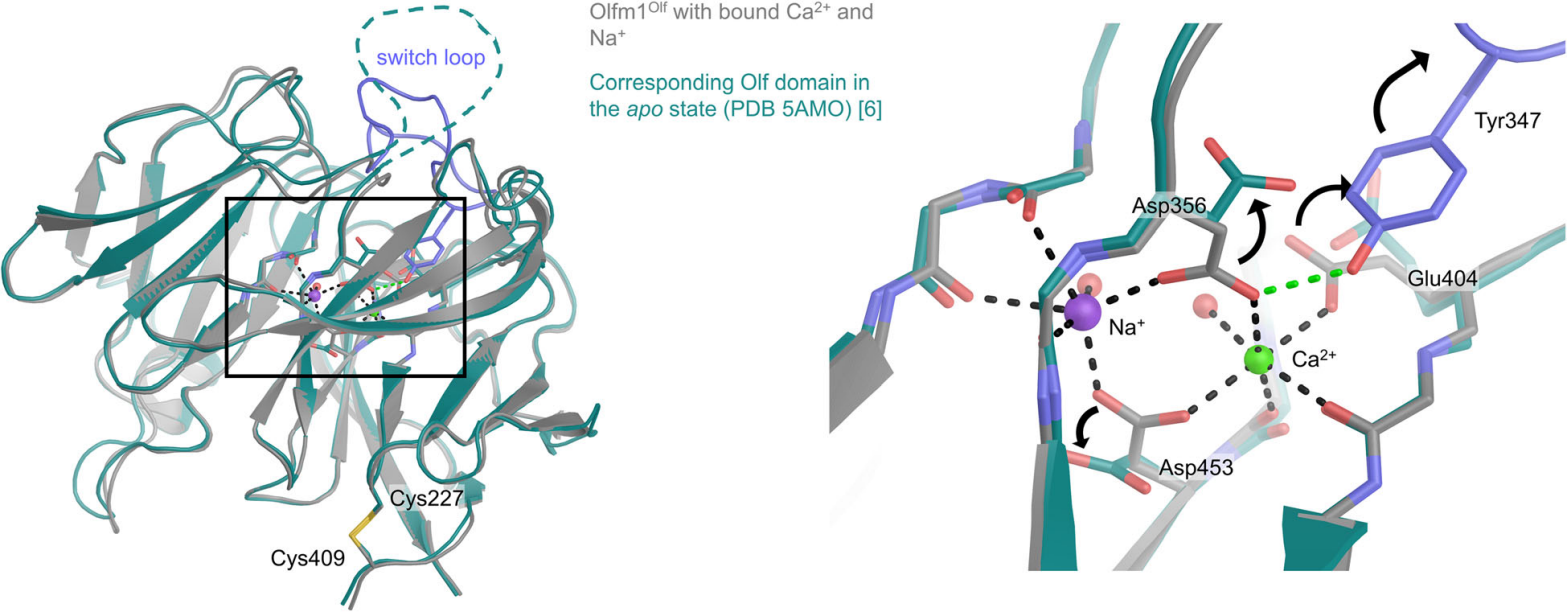
Comparison of Olfm1^Olf^ domain in the Ca^2+^- and Na^+^-bound state in grey with the previously-published *apo* state in teal [6]. The switch loop (violet) is only resolved in the Ca^2+^- and Na^+^-bound state. In the *apo* state, the negatively-charged side chains of Asp356, Glu404 and Asp453 are pushed outward in the absence of compensating positive charges from the Na^+^ and Ca^2+^ ions (indicated by arrows in the right panel). This most likely destabilises the conformation of the switch loop (violet) via Tyr347, which consequently is unstructured (indicated by a dashed line in the left panel) and thus not observed in the electron density of the *apo* form [6]. The intra-chain disulfide between Cys227 and Cys409 is shown in stick representation

**Fig. 6:**
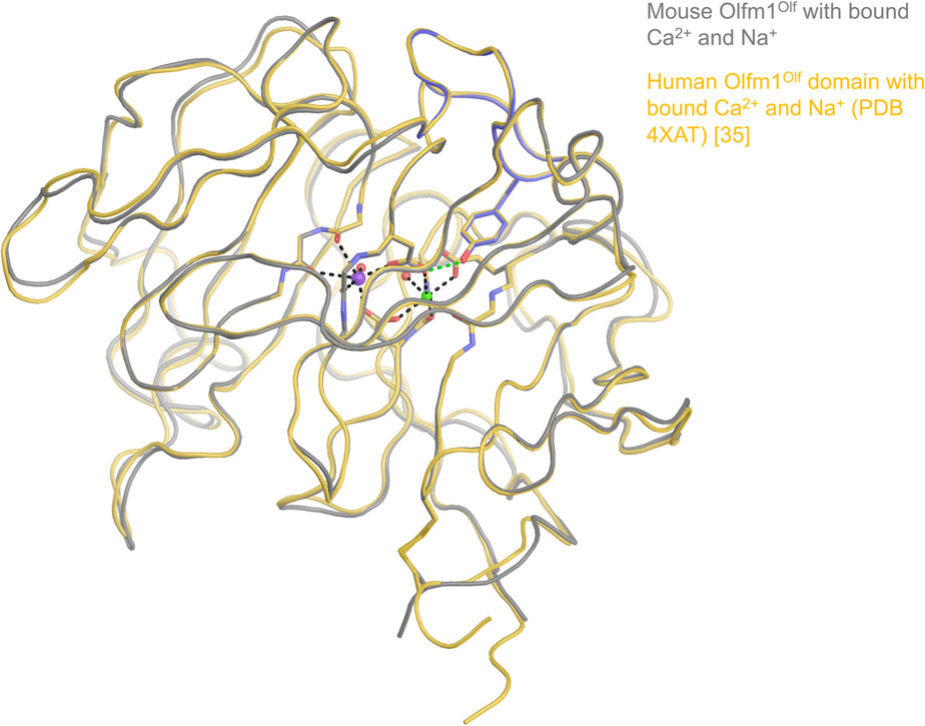
Mouse Olfm1^Olf^ and human Olfm1^Olf^ are very similar. Structural comparison of mouse Olfm1^Olf^ produced in mammalian (HEK293) cell line (grey) with human Olfm1^Olf^ produced in a bacterial expression system (PDB 4XAT, yellow) [35], both with bound Na^+^ and Ca^2+^, shows a high degree of similarity (C_α_ RMSD of 0.44). The switch loop (violet) is stabilised by bound Ca^2+^ and Na^+^ ions in both structures

We determined a high-resolution crystal structure of Endo-H-deglycosylated Olfm1^Olf^ (Fig. 4), which leaves a single acetylglucosamine (GlcNAc) attached to glycosylated asparagines. The deglycosylation step often aids crystallisation. Our best crystal diffracted to 1.25 Å resolution (Table 1), representing the highest resolution crystal structure of an Olfactomedin domain to date. The highly conserved monomeric Olf domain has a five-bladed β-propeller fold with a central metal ion binding site [29–34, 50]. The structure shows a high degree of similarity to the Olf-domain dimer in our previously-determined structure of a dimeric Olfm1^coil-Olf^ (PDB 5AMO) [6] (C_α_ RMSD of 0.55 Å, Fig. 5). A human Olfm1 Olf domain structure derived from bacterially-expressed protein [35] has a very similar structure to our mouse Olfm1^Olf^ (C_α_ RMSD of 0.44 Å, Fig. 6), other than it lacking N-linked glycosylation as a result of the expression system. Comparing the structures of mouse Olfm1^Olf^ and human Olfm1^Olf^ [35] with that of Olfm1^coil-Olf^, reveals structural rearrangements arising from the coordination of the Ca^2+^ and Na^+^ ions, that are not present in our previously determined structure of Olfm1^coil-Olf^ [6].

**Table 1:** Data collection and processing statistics

Data Collection		
PDB ID	6QHJ (Olfm1^Olf^)	6QM3 (Olfm1^coil-Olf^)
Beamline	ESRF ID30A-3	DLS I03
Resolution range (Å)^a^	36.76–1.25 (1.29–1.25)	75.99–1.89 (2.10–1.89)
Wavelength (Å)	0.9677	0.9762
Space Group	*I222*	*C*2
Unit Cell Dimensions		
a, b, c (Å)	61.79, 79.63, 111.72	160.7, 47.0, 105.0
α, β, γ	90.0, 90.0, 90.0	90.0, 117.4, 90.0
Measured reflections	416,910	95,762
Unique reflections	72,028	29,117
Mean I/σ	13.7 (1.0)	10.3 (1.7)
Completeness (%)	94.6 (63.9)	92.2 (77.2)
Redundancy	5.8 (2.4)	3.3 (3.4)
*R*_merge_ (%)	8.4 (90.8)	6.0 (75.1)
*CC*_1/2_	0.998 (0.445)	0.998 (0.551)
Data Refinement		
Resolution Range (Å)	33.75–1.25	71.30–2.00
Total reflections	72,027	28,629
Test set	3623	1459
*R*_work_	0.124	0.179
*R*_free_	0.141	0.216
No. of protein atoms	4374	4321
No. of ligand atoms	156	133
No. of water atoms	298	99
RMSD from ideal		
Bonds (Å)	0.010	0.007
Angles (°)	1.168	0.852
Mean *B* factor (Å^2^)	17.25	49.09
Ramachandran		
Favoured (%)	96.8	96.4
Outliers (%)	0.4	0.0
Clashscore^b^	1.11	3.58

^a^Numbers in parentheses correspond to values for the highest resolution shell

^b^Value calculated by MolProbity [49]

Because of the high resolution of our Olfm1^Olf^ diffraction data to 1.25 Å, we can unambiguously assign a Na^+^ and a Ca^2+^ ion bound in the central cavity of the β-propeller. Metal ion assignment was based on previous binding data [6, 35], coordination distances (Table 2) [51] and fit to the electron density. The Ca^2+^ ion is coordinated by the negatively-charged carboxyl groups of the side chains of Asp356, Asp453 and Glu404, as well as by the backbone carbonyl groups of Ala405 and Leu452 and a single water molecule (Fig. 4b and Table 2 for coordination distances and angles). The Na^+^ ion is also coordinated by the carboxylic acid groups of the side chains of both Asp356 and Asp453, as well as by the backbone carbonyl group of Leu357 and a different water molecule than the one coordinating the calcium ion (Fig. 4b, Table 2). In sum, three formal negative charges of the carboxylic acid groups of the side chains of Asp356, Asp453 and Glu404 are compensated by three formal positive charges of the bound Ca^2+^ and Na^+^ ions.

**Table 2:** Distances and angles of metal ion coordination in the Olfm1^Olf^ crystal structure

Distances:			Angles:			
		distance (Å)				angle (°)
Calcium	ASP356 O_(carboxylate)_	2.3	ASP356 O_(carboxylate)_	Calcium	GLU404 O_(carboxylate)_	86.7
Calcium	GLU404 O_(carboxylate)_	2.3	ASP356 O_(carboxylate)_	Calcium	ALA405 O_(carbonyl)_	93.1
Calcium	ALA405 O_(carbonyl)_	2.3	ASP356 O_(carboxylate)_	Calcium	ASP453 O_(carboxylate)_	105.5
Calcium	LEU452 O_(carbonyl)_	2.3	ASP356 O_(carboxylate)_	Calcium	H_2_O	94.1
Calcium	ASP453 O_(carboxylate)_	2.3	GLU404 O_(carboxylate)_	Calcium	ALA405 O_(carbonyl)_	83.0
Calcium	H_2_O	2.4	GLU404 O_(carboxylate)_	Calcium	LEU452 O_(carbonyl)_	83.7
			GLU404 O_(carboxylate)_	Calcium	H_2_O	82.7
Sodium	GLY302 O_(carbonyl)_	2.9	ALA405 O_(carbonyl)_	Calcium	LEU452 O_(carbonyl)_	89.2
Sodium	GLN303 O_(carbonyl)_	3.0	ALA405 O_(carbonyl)_	Calcium	ASP453 O_(carboxylate)_	103.8
Sodium	ASP356 O_(carboxylate)_	2.3	LEU452 O_(carbonyl)_	Calcium	H_2_O	81.4
Sodium	LEU357 O_(carbonyl)_	2.2	LEU452 O_(carbonyl)_	Calcium	ASP453 O_(carboxylate)_	83.6
Sodium	ASP453 O_(carboxylate)_	2.3	ASP453 O_(carboxylate)_	Calcium	H_2_O	88.4
Sodium	H_2_O	2.4				
			GLY302 O_(carbonyl)_	Sodium	GLN303 O_(carbonyl)_	66.6
ASP356 O_(carboxylate)_	TYR347 O_(hydroxyl)_	2.7	GLY302 O_(carbonyl)_	Sodium	ASP356 O_(carboxylate)_	88.5
			GLY302 O_(carbonyl)_	Sodium	LEU357 O_(carbonyl)_	78.3
			GLY302 O_(carbonyl)_	Sodium	H_2_O	98.6
			GLN303 O_(carbonyl)_	Sodium	LEU357 O_(carbonyl)_	88.4
			GLN303 O (carbonyl)	Sodium	ASP453 O_(carboxylate)_	95.8
			GLN303 O (carbonyl)	Sodium	H_2_O	71.0
			ASP356 O_(carboxylate)_	Sodium	LEU357 O_(carbonyl)_	101.4
			ASP356 O_(carboxylate)_	Sodium	ASP453 O_(carboxylate)_	109.4
			ASP356 O_(carboxylate)_	Sodium	H_2_O	99.9
			LEU357 O_(carbonyl)_	Sodium	ASP453 O_(carboxylate)_	98.5
			ASP453 O_(carboxylate)_	Sodium	H_2_O	77.8

Two non-bonding interactions appear to be formed between the backbone carbonyl groups of Gly302 and Gln303 with the Na^+^ ion that are at distances too great for direct coordination (2.9 and 3.0 Å, respectively) [52]. Furthermore, several of the coordination angles deviate substantially from 90 ° that may be expected for octahedral metal ion coordination and range from 81.4 to 105.5 ° for the Ca^2+^ ion and from 66.6 to 109.4 ° for the Na^+^ ion (Table 2). Still, the coordination of both Ca^2+^ and Na^+^ ions most closely resembles octahedral geometry (including the two non-bonding interactions with the backbone carbonyls of Gly302 and Gln303), rather than trigonal bipyramid, tetrahedral, square planar or square pyramid. Coordination distances range from 2.3 to 2.4 Å for the Ca^2+^ ion and from 2.2 to 2.4 Å for the Na^+^ ion, excluding the non-bonding interactions with the backbone carbonyls of Gly302 and Gln303. This is close to the ideal distances of 2.3 to 2.4 Å for the Ca^2+^ ion and of 2.3 to 2.5 Å for the Na^+^ ion [51].

A loop connecting propeller blade 2 to blade 3 (residues 339–352, sequence AGYNNMYHYAWGGH) that was unstructured in our previously-determined structure of Olfm1^coil-Olf^ (PDB 5AMO) [6] could now be fully observed in the electron density (Figs. 4 and 5), possibly as a result of a structural transition induced by Na^+^ and Ca^2+^ binding. Because of this structural transition, we will henceforward refer to this loop as the switch loop. The conserved residue Tyr347 stabilises the switch loop by forming a hydrogen bond between the Tyr347 hydroxyl group and the Asp356 carboxyl group (distance 2.7 Å) that plays a central role in coordinating both the bound sodium and calcium ions (Figs. 4 and 5). The Asp356 side chain adopts a different rotamer conformation and is pushed outward in the calcium-free structure [6]. Most likely this outward conformation arises from electrostatic repulsion by the other negatively-charged metal ion coordinating side chains, Glu404 and Asp453, that are no longer compensated by the positive charges of the Ca^2+^ and Na^+^ ions in the *apo* form. The outward Asp356 conformation in the *apo* form disrupts the hydrogen bond with Tyr347 and interferes with the conformation of the switch loop as observed in the Ca^2+^- and Na^+^-bound state by steric hindrance with Tyr347 (Fig. 5), possibly resulting in the switch loop being unstructured in the *apo* form.

The high-resolution crystal structure of Olfm1^Olf^ reveals a hydrophilic tunnel filled with water molecules running from the surface of the Olf domain to the metal ion binding sites (Fig. 4a and c). A similar water-containing tunnel was observed in the structure of the gliomedin Olf domain [29]. In Olfm1, the tunnel starts at the solvent-exposed top face between propeller blade 2 and 3 and runs between these two blades towards the metal ion binding sites. The tunnel proceeds further, almost to the bottom of the domain where it is closed by a hydrophobic plug made up of residues Pro253 in blade 1, Val359 in blade 3 and Pro456 in blade 5 (Fig. 4a). Twelve ordered water molecules are well resolved in this tunnel due to the high resolution of our electron density map, including the two water molecules involved in coordinating the Na^+^ and Ca^2+^ ions. The width of the tunnel varies along the pore axis with radii of 1.0 to 2.2 Å, as determined by the HOLE program [48] (Fig. 4a and c). These tunnel dimensions are too small to allow the passage of hydrated ions. The tunnel may, however, allow dehydrated metal ions, coordinated by tunnel-lining residues, to pass through this pore in the presence of thermal motion at physiological temperatures. Previous work showed that excess calcium stabilises folded purified Olfm1 [6, 35], whereas EDTA (a Ca^2+^ chelator) destabilises it [6]. In addition, sequestering of Ca^2+^ from the propeller domains by the excess of EDTA may increase the flexibility of the switch loop. This implies that calcium ions can dissociate from the folded protein, presumably by diffusion out of the β-propeller domain via the central tunnel.

### Dimeric Olfm1^coil-Olf^

A dimeric variant containing part of the coiled coil and Olf domain, corresponding to our previously crystallised proteolysis fragment, was designed with domain boundaries 212–478 (UNIPROT numbering, Fig. 1). This includes Cys221 that forms an inter-chain disulfide and covalently locks this construct in a dimeric form. The better-defined domain boundaries of this construct, compared to our previously described proteolysis fragment [6], provides more control over the quality of this sample. Only about a third of this recombinant Olfm1^coil-Olf^ sample formed disulfide-linked dimers as in the crystal structure, yet the monomeric and dimeric fractions could be separated from each other by two rounds of SEC (Figs. 1c and 3b, see Materials and methods section for details). Attempts to rescue the remaining monomeric fraction by reducing the disulfides and refolding in the presence of a redox couple were unsuccessful. The yields of dimeric Olfm1^coil-Olf^ are about 2 mg per litre of suspension HEK293 cell culture.

**Fig. 7:**
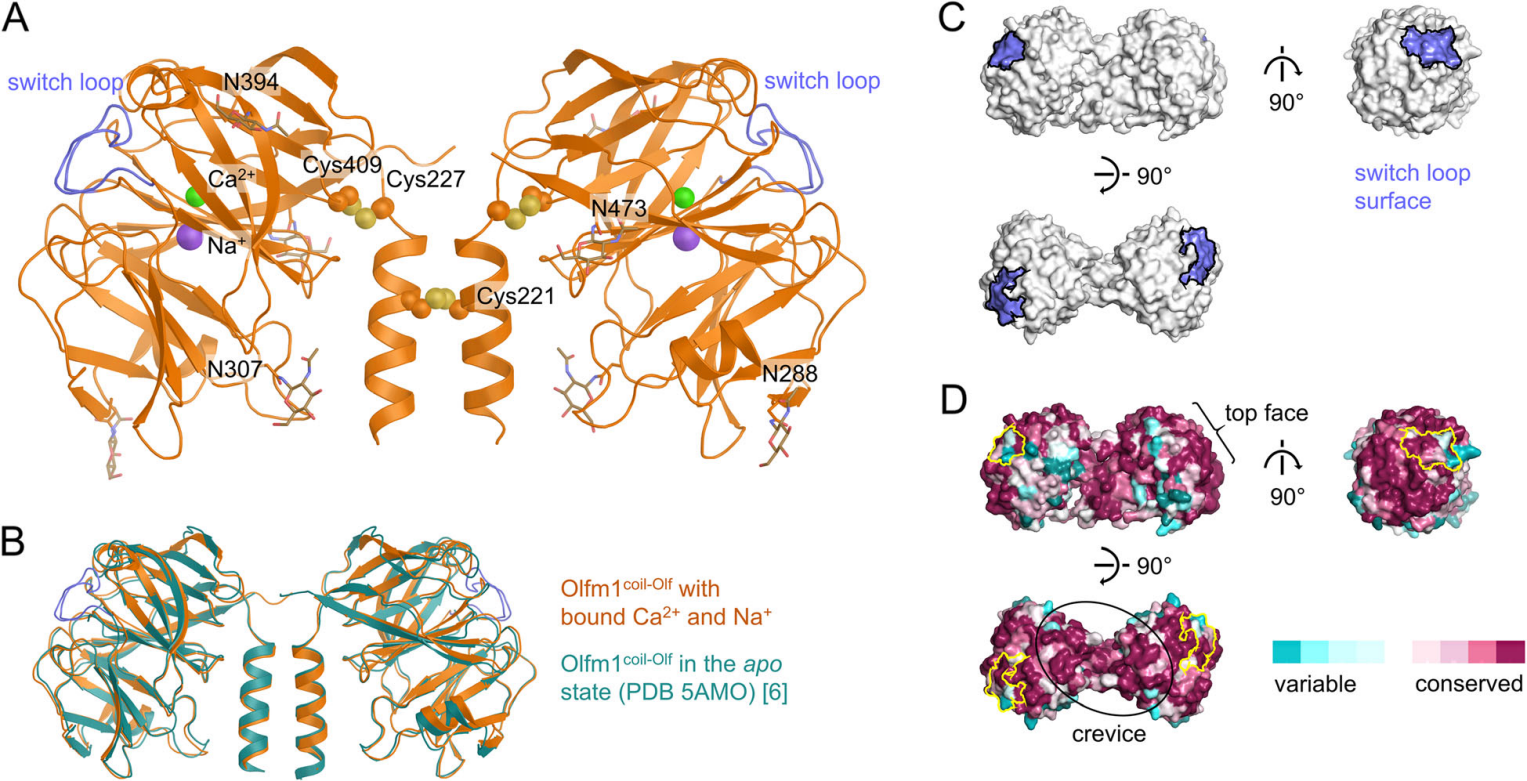
Crystal structure of dimeric recombinant Olfm1^coil-Olf^ with bound Na^+^ and Ca^2+^ ions. **a** Crystal structure of Olfm1^coil-Olf^ with bound Na^+^ (purple) and Ca^2+^ (green) and the switch loop in violet. Disulfides are indicated as spheres and N-linked glycans as brown sticks. **b** Comparison of the previously-solved limited proteolysis fragment of Olfm1^coil-Olf^ in the *apo* state (teal, PDB 5AMO) with the presented structure in the Na^+^- and Ca^2+^-bound state (orange) shows a very similar structure (C_α_ RMSD of 0.73 Å), except for the switch loop that is structured in the Na^+^- and Ca^2+^-bound state (it is unstructured in the *apo* state). **c** Surface representation of Olfm1^coil-Olf^ with the putative conditional interface formed by the switch loop (violet). **d** Conservation of Olfm1 amongst vertebrate orthologues plotted on the surface using Consurf [53]. The contour of the switch loop interface as in c is indicated with a yellow line. Both the top face of the β-propellers (indicated with an accolade in the left panel; front view in the right panel) and the crevice between the two Olf domains (indicated with an ellipse in the bottom panel) are highly conserved and may be interfaces for protein-protein interaction

We crystallised the dimeric form of Olfm1^coil-Olf^, after deglycosylation with Endo-H, with calcium chloride present in the buffer (Fig. 7a). Olfm1^coil-Olf^ crystallised in the same crystal form (space group *C*2) as the previously-determined limited proteolysis fragment corresponding to the same segment of Olfm1 (PDB 5AMO) [6], in spite of several differences such as the crystallisation condition, the crystallisation temperature, the presence of bound Ca^2+^ and Na^+^ ions and the glycosylation state. The Olfm1^coil-Olf^ structure reported previously [6] was not deglycosylated indicating that the glycans did not contribute substantially to the crystal packing. The crystals were not fully isomorphous and displayed subtle changes in unit cell parameters. The largest differences are in unit cell dimension b (47.0 Å in this study, whereas 43.9 Å in 5AMO) and angle β (117.4 ° in this study, whereas 114.2 ° in 5AMO). However, apart from the switch loop being observed in the calcium-stabilised conformation similar to the monomeric Olfm1^Olf^ structures discussed above, the structures of the limited proteolysis fragment and recombinantly-expressed Olfm1^coil-Olf^ were highly similar (C_α_ RMSD of 0.73 Å, Fig. 7b), validating the quality of the sample.

The outward-facing β-propeller top face of the Olf domain has been suggested to be involved in receptor binding [6] because of the conserved nature and the absence of N-linked glycans at this site. The switch loop forms part of this top face in the calcium-bound state and alters the properties of this surface. In the calcium-bound state the top face still consists of conserved residues and is devoid of glycans (Fig. 7c and d, right panel). By altering its conformation upon Ca^2+^ and Na^+^ binding, the switch loop might make this putative interface conditional, i.e. dependent on the presence of Ca^2+^ and/or Na^+^, as has been observed for the olfactomedin family member latrophilin3. Latrophilin3 binds to the cell surface receptor FLRT3 in a calcium-dependent way via the same loop [32]. Another putative region of interaction is the crevice between the two Olf domains, flanked by β-propeller blades 4 and 5 (Fig. 7d). This region is also conserved, void of N-linked glycans and the conformation of the switch loop is not affecting this area. Possibly, the top face of the Olf domains represents a Ca^2+^-dependent conditional interface, whereas the crevice between the two Olf domains is a putative Ca^2+^-independent interface for protein-protein interactions.

The Olfm1 paralogues Olfm2 and Olfm3, which share sequence identity conservation of 56.8–67.5% with Olfm1, have all elements responsible for a similar V-shaped disulfide-linked tetrameric arrangement as observed in Olfm1^BMZ^ (N-terminal cysteines, central coiled-coil domain, C-terminal cysteines and β-propeller domains) [6]. In the β-propeller domain, most of the sequence variation is, as expected, in the surface-exposed residues. The core of the protein is highly conserved and there are no insertions or deletions in this domain. One notable difference between these three paralogues is the (predicted) N-linked glycosylation pattern (Fig. 7a). Whereas the N-linked glycans on Olfm1 N307 and N473 are conserved in both Olfm2 and Olfm3, the one on N431 is only conserved in Olfm2 but not in Olfm3. The N-linked glycans on N288 and N394 on the other hand are not conserved in either Olfm2 or Olfm3. Other than that, Olfm2 is predicted to have a unique N-linked glycan on its β-propeller domain on N304 (UNIPROT numbering), which corresponds to Olfm1 N342, that is not part of an N-linked glycosylation motif. Olfm1 N342 is surface-exposed and resides in the switch loop. Whether N304 in Olfm2 is actually glycosylated has not been determined. As discussed in detail elsewhere [6, 35], the tertiary structure of the β*-*propeller domain of Olfm1 is very similar to that of the more distant homologues gliomedin, latrophilin3 and myocilin, yet the switch loop structure and the surface charge distribution of the β*-*propeller domain differ substantially.

### Tetrameric Olfm1^BMY^

We expressed a third construct based on the natural shorter BMY isoform (Olfm1^BMY^; UNIPROT residue 17–153, residue 153 being a glycine), that is expected to form disulfide-linked tetramers as it includes the NTT domain. Compared to the longer BMZ isoform, Olfm1^BMY^ lacks the C-terminal half of the coiled-coil domain as well as the C-terminal Olf domains (Fig. 1). This construct was expressed with reasonable yields (0.5 mg per litre of suspension HEK293 cell culture), albeit lower than the Olfm1^Olf^ or Olfm1^coil-Olf^ constructs. The non-reducing gel and SEC-MALS analysis support our prediction that this construct forms a disulfide-linked tetramer (Figs. 1c and 3d). Interestingly, while the light scattering by Olfm1^BMY^ clearly shows it to be tetrameric (determined mass of 76.8 ± 3.6 kDa from the MALS signal; expected 76.8 kDa for a tetramer), the elution volume corresponds to a much larger globular protein (eluting at the same volume as the calibration standard Aldolase; 158 kDa), suggesting Olfm1^BMY^ has an extended conformation. The observation that the tetrameric Olfm1^BMY^ constructs runs as a tetramer on non-reducing gel indicates that the three sets of cysteines in the NTT domain (Cys73, Cys75 and Cys85; Fig. 1a), which could form six disulfide bridges per tetramer, form interchain disulfides across the different sets of chains.

**Fig. 8:**
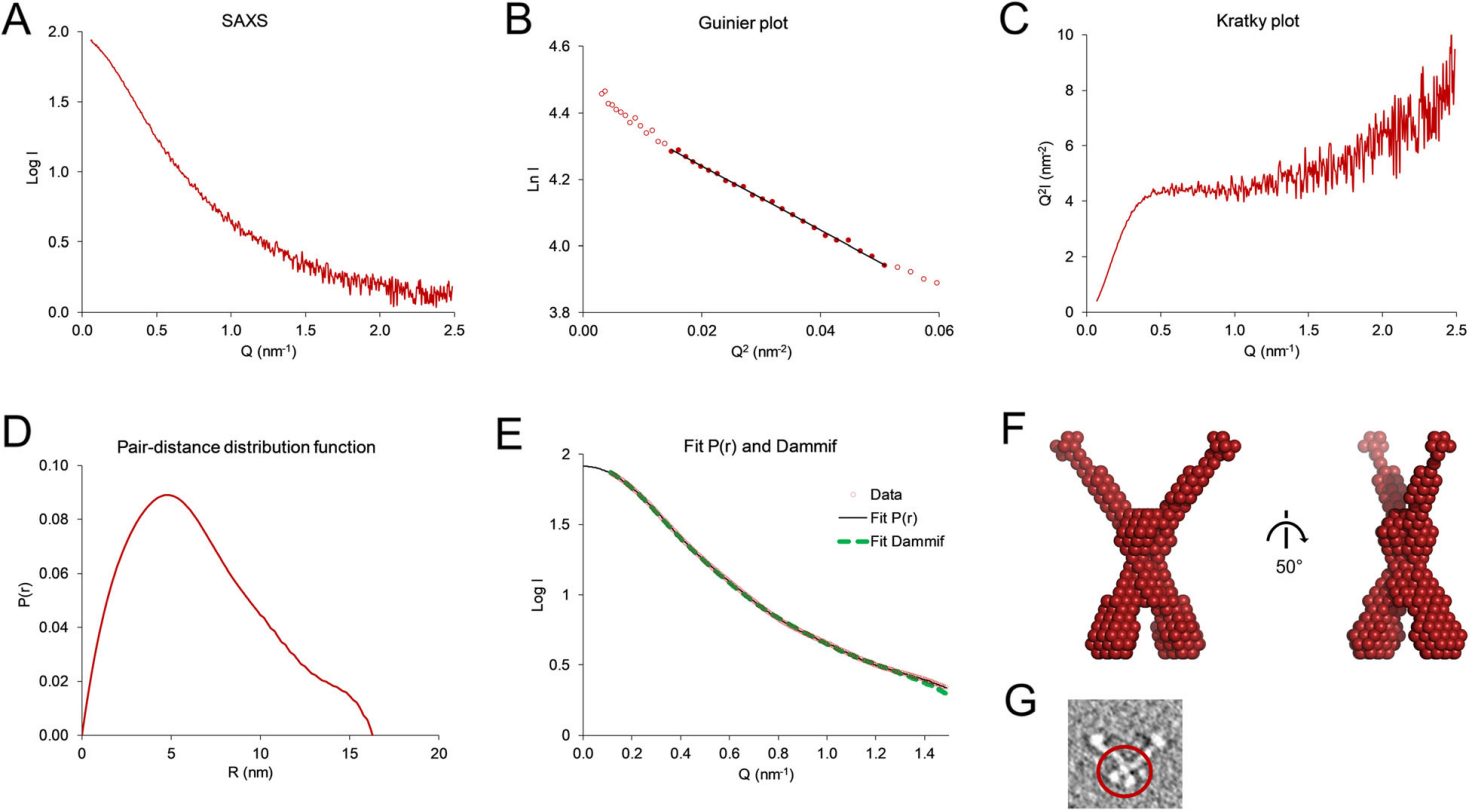
SAXS analysis indicates Olfm1^BMY^ is an “X”-shaped tetramer. **a** Averaged and reference-subtracted SAXS data of Olfm1^BMY^ at 0.615 mg/mL plotted as Log I vs Q. **b** Guinier plot of the SAXS data indicates Olfm1^BMY^ forms a tetramer (*R*_g_ of 5.4 nm and *I*_0_ corresponding to 84.0 kDa). **c** The Kratky plot suggests Olfm1^BMY^ has substantial flexibility yet is more structured than a random coil. **d** The pair-distance distribution function *P*(r) has an asymmetric bell shape with a maximum at 4.76 nm, and a maximum diameter (*D*_max_) of 16.3 nm. **e** The fit to the data (red circles) of the *P*(r) (black line) and the theoretical scattering of the ab-initio bead model by Dammif shown in panel f (green dashes) indicate a good agreement with the data (χ^2^ of 3.34 for the P(r) and 1.24 for the ab-initio model). **f** Dammif ab-initio model of Olfm1^BMY^ with enforced *C*2 symmetry reveals a shape similar to what was predicted based on the architecture of the longer BMZ isoform [6] (see panel g). **g** Previously-published slice through a negatively stained electron tomogram of Olfm1^BMZ^ [6], with the region corresponding to Olfm1^BMY^ indicated by a red ellipse

We did not succeed in obtaining crystals of Olfm1^BMY^, possibly due to the flexible nature of this protein segment. Therefore, we analysed the structure of Olfm1^BMY^ by SAXS, which provides structural information for proteins in the solution state. The SAXS *I*_0_ suggests a molecular mass of 84.0 kDa for Olfm1^BMY^ (Fig. 8, Table 3). This further supports Olfm1^BMY^ forming tetramers, although the value is higher than the theoretical mass confirmed by SEC-MALS of 76.8 kDa for a tetramer of Olfm1^BMY^ including 2 predicted N-linked glycans per chain. Guinier analysis of the SAXS data shows that Olfm1^BMY^ has a radius of gyration (*R*_g_) of 5.4 nm (Fig. 8b). The pair-distance distribution function *P*(r) has an asymmetric bell shape with a maximum at 4.76 nm, representing the most commonly occurring inter-atomic distance in the particle (Fig. 8d). The *P*(r) further shows Olfm1^BMY^ has a maximum dimension *D*_max_ of 16.3 nm and a Porod volume of 248 nm^3^. The Kratky plot indicates substantial flexibility, yet still more structure than a random coil (Fig. 8c).

**Table 3:** SAXS parameters of Olfm1^BMY^

SASBDB code	SASDF96
Concentration (mg/mL)	0.615
*R*_g_ (nm)	5.4
*I*_0_	84.0
*D*_max_ (nm)	16.3
Porod Volume (nm^3^)	248

Based on our previous work on the longer BMZ isoform of Olfm1 [6], we expected the Olfm1^BMY^ construct (that makes up the N-terminal part of the longer BMZ isoform) to have two-fold rotational symmetry (*C*2). Ab-initio modelling based on our SAXS data using the Dammif software [47] with enforced *C*2 symmetry yields models that closely mimic the shape of the Olfm1^BMZ^ N-terminal segment observed previously by negative stain electron tomography (Fig. 8f and g) [6]. Thus, the shift on non-reducing gel, the SEC-MALS analysis and the SAXS data support the notion that this natural isoform folds into tetramers with the same structure as the corresponding region within the longer BMZ isoform.

## Discussion

The obtained high-resolution crystal structures of the monomeric Olfm1^Olf^ and dimeric Olfm1^coil-Olf^ constructs in the calcium- and sodium-bound states yield several new insights. Similar to latrophilin [32], a surface loop of Olfactomedin-1 is stabilised by the internally-bound Ca^2+^ and Na^+^ ions in the core of the Olf domain. Ca^2+^ and Na^+^ ions would be able to bind the buried binding sites in the Olf domain during folding in the high calcium environment of the secretory pathway, also in the absence of any tunnel. The presence of this tunnel suggests these metal ion binding sites do not just serve a structural function. Rather, this tunnel might allow the Olf domain to sample the concentration of these ions in the microenvironment of Olfm1 beyond the ER. The Olf domain of Olfm1 could serve as a calcium sensor by providing a conditional interface for protein-protein interactions, the condition being the presence of Ca^2+^ at sufficiently high concentration. Since Olfm1 was found to be enriched in synapses and present in the synaptic cleft, it is tempting to speculate that synaptic activity, which causes substantial local decreases in extracellular calcium concentration [54] as a result of the opening of pre-synaptic voltage-gated calcium channels and post-synaptic NMDA receptors and calcium-permeable AMPA receptors, might be sampled by the Olf domains of Olfm1. The ability of the Olf domain to sample calcium concentration might serve to control short-term or long-term depression at synapses or prevent excitotoxicity as a negative feedback sensor. Further studies are required to determine if the equilibrium and kinetic dissociation constants (*K*_d_ and *k*_d_) for calcium binding are in the correct concentration and time regime to sample such physiological decreases in calcium concentration, and to elucidate what (if any) protein binding partners of Olfm1 are calcium-dependent. Interestingly, various trans-synaptic protein complexes have been shown to be depending on or stabilised by calcium ions, such as β-Neurexin-neuroligin [55, 56], β-Neurexin-LRRTM2 [57, 58], β-Neurexin-Cerebellin1-GluD2 [59], Cadherins [60, 61], and Latrophilin3-Flrt [32], suggesting this might be a more general mechanism.

In this regard, another attractive hypothesis that could be studied with our constructs would be that Olfm1 is involved in trans-synaptic interactions. Olfm1 binds directly to post-synaptic AMPA receptors [8] and has a V-shaped oligomeric architecture similar to the secreted protein Cerebellin1 [6, 59], which engages with the AMPA receptor homologue GluD2 in the synaptic cleft of specific synapses [59, 62, 63]. Cerebellin1 tethers these GluD2 receptors to pre-synaptic β-Neurexin [59, 63]. AMPA receptors where recently found to be aligned on a subsynaptic scale to pre-synaptic vesicle release sites [64]. The similar architecture of Olfm1 and Cerebellin1 suggests Olfm1 might bind to AMPA receptors in a similar manner as Cerebellin1 to GluD2 receptors.

A putative pre-synaptic anchor for Olfm1 (analogous to β-Neurexin for Cerebellin-1) could be APP, a single-pass transmembrane protein that is known to be associated with the pre-synaptic vesicle release machinery [65, 66]. APP was found previously to interact directly with Olfm1, thereby modulating the proteolytic processing of APP by secretase enzymes [13]. The tetrameric nature, shape and dimensions of Olfm1^BMZ^ might allow it to engage multiple pre- and post-synaptic receptors, benefitting from surface avidity similar to for example IgGs [67]. Further research is required to determine if Olfm1 can simultaneously engage both pre-synaptic APP and post-synaptic AMPA receptors. If Olfm1 does trans-synaptically tether AMPA receptors to APP in vivo, this could potentially have implications for Alzheimer’s disease, as mutations affecting the proteolytic processing of APP (both in APP itself and its secretases) are well-known to cause early-onset Alzheimer’s disease [68, 69], the early phase of which is characterised by a loss of AMPA receptors and synaptic malfunctioning [70–72].

## Conclusions

Here we present three novel constructs of Olfm1; one monomeric (Olfm1^Olf^), one dimeric (Olfm1^coil-Olf^) and one tetrameric (Olfm1^BMY^), with optimised expression and purification strategies. We validated our expression and purification strategies by analysing the samples by (non)-reducing SDS-PAGE and analytical SEC-MALS. Furthermore, we determined high-resolution crystal structures for the monomeric and dimeric constructs, and characterised the size and shape of the tetrameric Olfm1^BMY^ construct by SAXS. These constructs, in combination with our previously-published tetrameric BMZ construct [6], will allow the precise probing of interactions of binding partners with specific domains of Olfm1, and can be used in functional assays to study Olfm1 in the (mature) mammalian brain.

The constructs and purification strategies presented here could be used to find domain-specific and calcium-dependent binding partners. For example, the protein samples could be used as probes to identify novel Olfm1 interactors by proteomic screening of interaction partners pulled down by Olfm1-functionalised beads from brain lysate in the presence of either calcium or EDTA. Both new and established interactions could be further quantified and assigned to specific domains by direct binding assays such as surface plasmon resonance and isothermal titration calorimetry with the described constructs. These new constructs, together with our previously published tetrameric Olfm1^BMZ^ construct, could also be used for in vitro cell or bead clustering assays to test if pre/post-synaptic receptor binding *in trans* is supported by Olfm1. Moreover, the Olfm1^BMY^ and Olfm1^coil-Olf^ constructs could be used as acute dominant negatives by interfering with one interaction but not linking to a third protein in functional assays, such as electrophysiological determination of synaptic input/output ratio or long-term potentiation, or morphological characterisation of synapses and dendritic spines, for example in combination with knockout or knockdown animals [22]. In conclusion, there are many exciting hypotheses about the functions of Olfm1 in the mature (mammalian) brain that remain to be tested, for which our novel constructs and their associated purification strategies might provide a starting point.

## Data Availability

The atomic coordinates and structure factors (codes 6QHJ and 6QM3 for Olfm1^Olf^ and Olfm1^coil-Olf^, respectively) have been deposited in the Protein Data Bank (http://wwpdb.org/). The SAXS data and models of Olfm1^BMY^ have been deposited in the small angle scattering databank (https://www.sasbdb.org/) with accession code SASDF96.

## Abbreviations

AMPA: α-amino-3-hydroxy-5-methyl-4-isoxazolepropionic acid
APP: Amyloid precursor protein
DLS: Diamond light source
ESRF: European Synchrotron Radiation Facility
GntI: *N*-acetylglucosaminyltransferase I
MWCO: Molecular weight cut-off
NMDA: N-Methyl-D-aspartic acid
NTT: N-terminal tetramerisation domain
Olf: Olfactomedin
RMSD: Root mean square deviation
SAXS: Small-angle X-ray scattering
